# Immune Checkpoint Inhibitors for Genitourinary Cancers: Treatment Indications, Investigational Approaches and Biomarkers

**DOI:** 10.3390/cancers13215415

**Published:** 2021-10-28

**Authors:** Brian W. Labadie, Arjun V. Balar, Jason J. Luke

**Affiliations:** 1 Division of Hematology/Oncology, Department of Medicine, Vagelos College of Physicians and Surgeons, Columbia University, New York, NY 10032, USA; bl2879@cumc.columbia.edu; 2 Perlmutter Cancer Center, NYU Langone Health and New York University, New York, NY 10016, USA; arjun.balar@nyulangone.org; 3 UPMC Hillman Cancer Center, University of Pittsburgh, Pittsburgh, PA 15232, USA

**Keywords:** immunotherapy, immune checkpoint inhibitors, genitourinary cancer, kidney, prostate, testicular, bladder cancer

## Abstract

**Simple Summary:**

Immune checkpoint inhibitors (ICI) have reshaped treatment paradigms of multiple solid organ malignancies. In genitourinary malignancies (GU), ICI provide significant clinical benefit and are approved for use in localized and metastatic renal cell carcinoma and urothelial carcinoma. Investigational approaches to maximize clinical benefit and expand use of ICI across GU malignancies are actively being pursued. In addition, biomarkers predictive of clinical benefit to ICI have been identified; however, further validation and incorporation into guideline-based management remain active areas of investigation.

**Abstract:**

Cancers of the genitourinary (GU) tract are common malignancies in both men and women and are a major source of morbidity and mortality. Immune checkpoint inhibitors (ICI) targeting CTLA-4, PD-1 or PD-L1 have provided clinical benefit, particularly in renal cell and urothelial carcinoma, and have been incorporated into standard of care treatment in both localized and metastatic settings. However, a large fraction of patients do not derive benefit. Identification of patient and tumor-derived factors which associate with response have led to insights into mechanisms of response and resistance to ICI. Herein, we review current approvals and clinical development of ICI in GU malignancies and discuss exploratory biomarkers which aid in personalized treatment selection.

## 1. Introduction

Cancers of the genitourinary (GU) tract are a major source of cancer morbidity and mortality. GU malignancies include prostate adenocarcinoma, the most common malignancy in men, urothelial and renal cell carcinoma (RCC), the sixth and eighth most common cancer among men and women combined, and testicular cancer, the most common solid tumor malignancy among men younger than 35 years old [1]. There have been substantial improvements in therapeutic strategies for treatment of these malignancies in the recent past. Current standard of care includes diverse treatment modalities such as hormonal therapy in prostate cancer, chemotherapy for urothelial and testicular cancer and targeted treatment of vascular–endothelial growth factor and mammalian target of rapamycin (mTOR) signaling in RCC.

Therapies which harness anti-cancer immune responses, referred to broadly as immunotherapy, have changed the paradigm of treatment for many cancers in recent years. Immune checkpoint inhibitors (ICI) are a class of immunotherapy which block immune-inhibitory receptors, or immune checkpoints, and re-invigorate anti-cancer immunity and facilitate tumor elimination [2]. Within the last decade, ICI which target CTLA-4, PD-1 or PD-L1 have achieved profound and durable responses in many patients and have been approved for use in multiple cancers (Table 1) [3]. Herein, ICI will refer to anti-CTLA-4 and anti-PD-1/PD-L1 agents unless otherwise noted. In GU malignancies, most notably in RCC and urothelial cancer, ICI as monotherapy or in combination with other agents have emerged as FDA approved standards of care in the treatment of metastatic disease as well as in the curative adjuvant and neoadjuvant localized disease settings [4,5,6,7,8,9,10]. 

Unfortunately, practice-changing efficacy of ICI has been limited to a subset of tumor types, such as melanoma, kidney, bladder and non-small cell lung cancer (NSCLC), and many tumors fail to derive benefit, such as tumors of the gastrointestinal tract [3]. Features of tumor-immune biology have been found to associate with response and resistance to ICI and are being evaluated in the clinical setting for use as predictive biomarkers. For example, increased PD-L1 expression on tumor cells correlates with clinical benefit to anti-PD-1/PD-L1 ICI in patients with NSCLC, a trend that is observed in some but not all ICI-responsive malignancies [11]. Patients without threshold PD-L1 expression achieve responses, however, suggesting PD-L1 assessment alone is insufficient to identify patients for whom ICI should be withheld [11,12]. Biomarkers which act as surrogates of T cell infiltration, also described as the T cell-inflamed tumor microenvironment (TME), have been observed to associate with improved outcomes to ICI across multiple cancer types [2,13,14,15]. In addition, high non-synonymous somatic tumor mutational burden (TMB) and genomic insertions and deletions correlate with benefit to ICI [16,17,18]. Patient demographic and clinical parameters such as the commensal microbiome and body-mass index have also been shown to associate with responses to ICI [19,20,21,22]. Elucidating a mechanistic basis for these associations are areas of active research.

**Figure 1:**
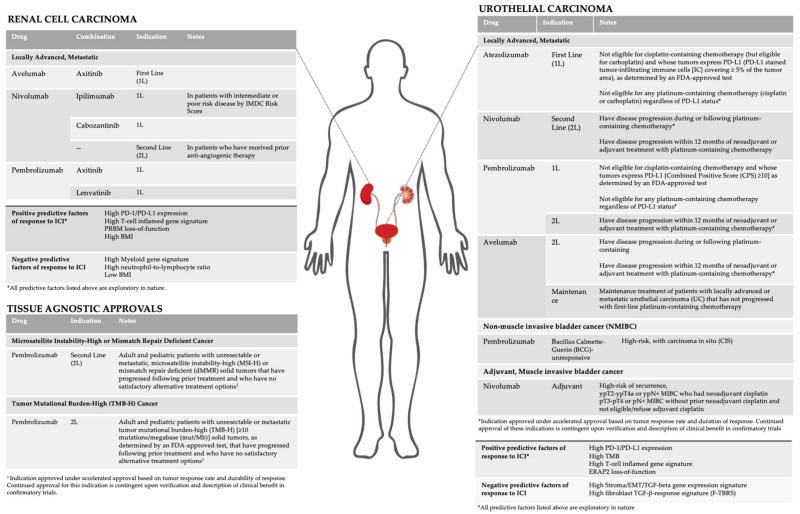
Current indications and biomarkers for immune checkpoint inhibitors in GU malignancies.

Herein, we review current FDA approved indications and active areas of clinical development of ICI in GU malignancies (Figure 1). In addition, we discuss investigational biomarkers and their association with response to ICI.

## 2. Treatment Indications, Investigational Approaches and Biomarkers of ICI in GU Cancers

### 2.1. Kidney Cancer (Renal Cell Carcinoma)

Renal cell carcinomas (RCC) are the eighth most common cancer diagnosis with an estimated 74,000 new cases diagnosed in 2019 [23]. RCC is subclassified into two groups: clear cell histology which makes up 75–80% of cases and non-clear cell histology which includes papillary, sarcomatoid, chromophobe subtypes [24]. Targeted therapies with vascular endothelial growth factor (VEGF) tyrosine kinase inhibitors (TKIs), mammalian target rapamycin (mTOR) inhibitors are widely used in first and second-line treatment in both metastatic clear cell and non-clear cell RCC [25,26]. In addition, clear cell RCC was one of the first cancers to be treated with cytokine-based immunotherapy such as recombinant IFN-⍺ and high-dose IL-2 [27,28]. High-dose IL-2 maintains a category 2B recommendation for treatment of metastatic disease in certain circumstances by the National Comprehensive Cancer Network (NCCN). More recently, clinical trials have established a role for ICI and ICI-based combinations in multiple settings for the treatment of clear cell and non-clear cell RCC. Results of these trials are summarized in Table 2.

#### 2.1.1. ICI Indications

ICI is approved for the treatment of untreated and refractory metastatic clear cell RCC. ICI use in the adjuvant and neoadjuvant setting are in advanced stages of clinical development. In metastatic disease, treatment guidelines and FDA indications take into account International Metastatic RCC Database Consortium (IMDC) Risk groups, which assign a risk score based on clinical variables such as time from diagnosis to systemic therapy greater than or less than 12 months, performance status, hemoglobin, neutrophil and platelet counts and corrected calcium level. Patients without any positive variables are considered favorable-risk, while those with one or two positive variables are considered intermediate-risk, and those with three or more factors considered poor-risk [27,29].

In localized disease, neoadjuvant and adjuvant ICI is being investigated and treatment considers risk groups based on histology, grade and TNM stage. Subsequent sections referring to clinical trials and indications for RCC discuss clear cell histology and non-clear cell histology separately.

##### Advanced/Metastatic Clear Cell RCC

*First Line*

In patients with previously untreated metastatic clear cell RCC, treatment indications are based on IMDC risk group. Combination anti-PD-1/PD-L1 plus VEGF TKI is approved in all risk groups while combination nivolumab plus ipilimumab is indicated in patients with intermediate- and poor-risk disease.

Nivolumab plus ipilimumab was approved for first-line therapy based on the phase 3 CheckMate-214 trial versus sunitinib monotherapy [7]. In this trial, nearly 90% of 1096 intention-to-treat population had either intermediate or poor-risk disease, and in these patients, treatment with nivolumab plus ipilimumab resulted in superior 18-month OS. In favorable-risk patients, treatment with nivolumab plus ipilimumab resulted in inferior PFS and OS than sunitinib monotherapy, results which were confirmed at 42-month follow-up analysis [30]. As such, nivolumab plus ipilimumab is not indicated for first-line treatment in patients with favorable-risk disease at time of diagnosis.

Multiple anti-PD-1/PD-L1 plus VEGF TKI combinations have been approved for first-line treatment of advanced RCC regardless of risk group. These combinations include axitinib plus pembrolizumab evaluated in the KEYNOTE-426 clinical trial, lenvatinib plus pembrolizumab evaluated in the CLEAR clinical trial, axitinib plus avelumab evaluated in the JAVELIN Renal 101 clinical trial and cabozantinib plus nivolumab evaluated in the CheckMate-9ER clinical trial [4,6,31,32]. Across all studies, combination treatment resulted in a higher ORR, median PFS, OS and a higher percentage of complete responses than sunitinib monotherapy. Extended follow-up results have confirmed these findings [5,33,34]. 

Despite clear activity of anti-PD-1/PD-L1 plus VEGF TKI combinations, it is unclear if the activity is synergistic or additive. VEGF inhibitors have been shown to normalize tumor vasculature and increase immune cell tumor infiltration, decrease immunosuppressive cell populations and promote T cell priming and activation via dendritic cell maturation [35,36,37]. Interestingly, the phase 3 IMMotion151 trial of atezolizumab plus bevacizumab, a monoclonal antibody to VEGF receptor, did not improve overall survival, calling into question whether targeted VEGF blockade alone, in contrast to the multi-kinase inhibition of VEGF TKIs, is sufficient to enhance ICI [38,39].

In patients with intermediate- and poor-risk disease, evidence-based selection of first-line treatment between nivolumab plus ipilimumab or anti-PD-1/PD-L1 plus VEGF TKI combinations remains undefined. Longer follow-up is required to determine if combination anti-PD-1/PD-L1 plus VEGF-TKI will produce as durable of complete responses as nivolumab plus ipilimumab since median OS has not been reached in many pivotal studies of anti-PD-1/PD-L1 plus VEGF TKI. These outcomes will provide basis for comparison and facilitate discussion with patients when selecting treatment; however, cross-study comparison has limitations. In the long-term, a head-to-head phase 3 clinical trial comparing these two strategies is warranted.

ICI-specific outcome measures such as treatment free survival (TFS), defined as time from ICI cessation to subsequent systemic therapy initiation or death, should be considered in future clinical trials [38]. In favorable-risk patients in the CheckMate-214 trial, despite inferior outcomes to sunitinib monotherapy, patients treated with nivolumab plus ipilimumab achieved higher rates of complete response and duration of TFS [30]. These observations represent important patient-centered metrics that are critical to assess in future trials [39].

*Second Lin* *e*

In current practice, most patients receive upfront anti-PD-1 in combination with either anti-CTLA-4 agent or VEGF TKI, and there is limited data to guide subsequent treatment selection. Investigators have begun to conduct clinical trials which specifically enroll patients who have progressed on anti-PD-1/anti-PD-L1 based combination as most recent therapy. In patients with metastatic RCC initially treated with anti-PD-1 therapy plus VEGF TKI, salvage nivolumab plus ipilimumab has demonstrated activity and is approved regardless of IMDC risk group [40,41,42]. A recent phase 1b/2 clinical trial in advanced solid tumors including RCC demonstrated encouraging anti-tumor activity for lenvatinib plus pembrolizumab for post-ICI treatment [43]. The role of continuing the anti-PD-1/PD-L1 backbone in post-ICI treatment is being evaluated in the CONTACT-03 trial of atezolizumab plus cabozantinib versus cabozantinib alone in patients with metastatic RCC after progression on anti-PD-1/PD-L1 treatment (NCT04338269).

In patients treated first-line with VEGF TKI monotherapy, nivolumab monotherapy is approved based on results from the phase 3 CheckMate 025 trial which demonstrated improved OS compared with everolimus [44,45].

*Non-clear cell RCC*

Analysis of the CheckMate 374 study of nivolumab monotherapy in previously treated RCC revealed meaningful responses in non-clear cell RCC [46]. The KEYNOTE-427 trial Cohort B, a phase 2 trial of pembrolizumab monotherapy in 165 patients with non-clear cell RCC, demonstrated encouraging anti-tumor activity, particularly in papillary or unclassified histology [47]. A multi-center phase 2 trial demonstrated efficacy of atezolizumab in combination with bevacizumab with ccRCC or non-clear cell RCC with >20% sarcomatoid differentiation [48]. A phase 2 trial of nivolumab plus cabozantinib demonstrated responses in non-clear cell RCC however activity in chromophobe was limited [49]. Nivolumab plus ipilimumab versus standard of care (VEGF TKI) in patients with previously untreated and advanced non-clear cell RCC demonstrated substantially higher ORR and is currently being evaluated in an ongoing multi-center phase 2 trial (SUNNIFORECAST) [50]. 

**Table 2:** ICI clinical trials in renal cell carcinoma.

ICI Agent	Trial (Phase)	Setting	Line	Arms	N	ORR, CR, PR	Median PFS (Mo) (95% CI)	PFS HR (95% CI)	Overall Survival	OS HR (95% CI)	OS in PD-L1 Positive	AE
Nivolumab	CheckMate 214 (3) NCT02231749 [7,30]	RCC, metastatic Intermediate/Poor IMDC risk groups	1	Nivolumab + ipilimumab	550	42%, 9%	11.2		48.1 mo (35.6–NE)	0.65 (0.54–0.78)		Any grade: 93% G3-4: 46%
				Sunitinib	546	27%, 1%	8.3		26.6 mo (22.1–33.5)			Any grade: 97% G3-4: 63%
Nivolumab	CheckMate 9ER (3) NCT03141177 [5]	RCC, metastatic All risk groups	1	Nivolumab + cabozantinib	323	55.7%, 8%, 47.7%	16.6 (12.5–24.9)	0.51 (0.41–0.64; *p* < 0.001)	NE	0.60 (0.40–0.89; *p* = 0.001)		Any grade: 96.6% G3: 60.6%
				Sunitinib	328	27.1%, 4.6%, 22.6%	8.3 (7.0–9.7)		NE (22.6-NE)			Any grade: 93.1% G3: 50.9%
Nivolumab	CheckMate 025 (3) NCT01668784 [44]	RCC, metastatic All risk groups	2	Nivolumab	406	25%, 1%, 24%	4.6 (3.7–5.4)	0.88 (0.75–1.03; *p* = 0.11)	25 mo 95% CI (21.8-NE)	0.73 98.5% CI 0.57–0.93 *p* = 0.002)		G3–4: 19%
				Everolimus	397	5%, <1%, 5%	4.4 (3.7–5.5)		19.6 mo (17.6–23.1)			G3–4: 37%
Nivolumab	CheckMate 009 (1) NCT01358721 [51]	RCC, metastatic	2	Nivolumab	92	15%, 2%, 12%			16.4 mo (10.1-NR) in previously treated pts 25.2 mo (12.0-NR) in untreated pts		23.4 mo in pts with <5% PD-L1+ tumor expression NR with >5% PD-L1 tumor expression	
Pembrolizumab	KEYNOTE-426 (3) NCT02853331 [4,33]	RCC, metastatic All risk groups	1	Pembrolizumab + axitinib	432	59.3%, 5.8%, 53.5%	15.1 (12.6–17,7)	0.69 (0.57 to 0.84; *p* < 0.001)	NE	0.53 (0.38–0.74; *p* < 0.0001)		Any grade: 96% >/= G3 : 67%
				Sunitinib	429	35.7%, 1.9%, 33.8%	11.1 (8.7–12.5)		35.7 (33.3-NE)			Any grade: 98% >/= G3 : 62%
Pembrolizumab	CLEAR (3) NCT02811861 [34]	RCC, metastatic All risk groups [34]	1	A Pembrolizumab + Lenvatinib	355	71.0%, 16.1%, 54.9%	23.9 (21–28)	A vs. C 0.39 (0.32–0.49; *p* < 0.0001)	NR (33.6-NE)	A vs. C 0.66 (0.49–0.88; *p* < 0.004)		>/= G3 : 82.4%
				B Everolimus + lenvatinib	357	53.5%, 9.8%, 43.7%	14.7 (11.1–16.7)	B vs. C 0.65 (0.53–0.8; *p* < 0.001)	NR (NE-NE)	B vs. C 1.15 (0.88–11.5; *p* = 0.3)		>/= G3 : 83.1.%
				C Sunitinib	357	36.1%, 4.2%, 31.9%	9.2 (11–17)		NE (NE-NE)			>/= G3 : 71.8%
Pembrolizumab	KEYNOTE-427 (2) [52]	RCC, metastatic	1	Pembrolizumab	107	36.4 (3.6%,32.7%	7.1 (5.6–11.0)		NR			>/= G3 : 30%
Pembrolizumab	KEYNOTE-427 (Cohort B) (2) [47]	Non-ccRCC, metastatic	1	Pembrolizumab	165	24.8% (4.8%, 20%)	4.2 (2.9–5.6)		28.9 (24.3-NR)			Any grade: 69.7%
Avelumab	Javelin Renal 101 (3) NCT02684006 [6]	RCC, metastatic All risk groups	1	Avelumab + axitinib	442	51.4%, 3.4%, 48.0%	13.3 (11.1-NE)	0.69 (0.574–0.825); *p* < 0.0001;	Median not reached	HR 0.65 (0.413–0.933)	PD-L1+: Not reached	>/= G3: 71.2%
				Sunitinib	444	25.7%, 1.8%, 23.9%	8.4 (6.9–11.1)		Median not reached		PD-L1+: 28.6 (27.4–NE) HR: 0.83 (95% CI 0.596–1.151), 1-sided *p* = 0.1301	>/= G3: 71.5%
Avelumab	JAVELIN Solid Tumor (1b) NCT01772004 [53]	RCC, metastatic All risk groups	1 or 2	Avelumab	82 (62 in 1L, 20 in 2L	1L: 16.1%, 1.6%, 14.5% 2L: 10%, 0%, 3%	1L: 8.3 (5.5–9.5) 2L: 5.6 (2.3–9.6)		1L: NE 2L: 16.9 (8.3-NE)			G3–4 1L: 12.9% 2L: 5%
Atezolizumab	IMMotion151 (3) NCT02420821 [54]	RCC, metastatic All risk groups	1	Atezolizumab + bevacizumab	454	37%, 5%, 31%	11.2	0.74 [95% CI 0.57–0.96]; *p* = 0.0217	33.6 (29.0-NE)	0.93 (0.76–1.14)	OS in PD-L1+: 34.0 (28.6-NE)	G3–4: 40%
				Sunitinib	461	33%, 2%, 31%	7.7		35.9 (27.9-NE)		32.7 (23.3-NE) HR: 0.84 (0.62–1.15; *p* = 0.2587)	G3–4: 54%
Atezolizumab	IMmotion150 (2) NCT01984242 [55]	RCC, metastatic All risk groups	1	A Atezolizumab + bevacizumab	101	32%, 7%, 25%	11.7 (8.4–17.3)	A vs. C 1.00 (0.69–1.45; *p* = 0.982)			PFS in PD-L1+ A vs. C 0.64 (0.38–1.08; *p* = 0.095)	G3–4: 63%
				B Atezolizumab	103	25%, 11%, 14%	6.1 (5.4–13.6)	B vs. C 1.19 (0.82–1.71; *p* = 0.358)			B vs. C 1.03 (0.63–1.67; *p* = 0.917)	G3–4: 40%
				C Sunitinib	101	29%, 5%, 24%	8.4 (7.0–14.0)					G3–4: 69%
Atezolizumab	(1a) [56]	RCC, metastatic	1 or 2	Atezolizumab	77	15%	5.6 (3.9–8.2)		28.9 (20-NR)			G3: 21%
PERIOPERATIVE												
ICI Agent	Trial (Phase)	Setting	Line	Arms	N	Disease Free Survival (DFS)		Median OS				AE
	KEYNOTE-564 (3) [8]	RCC, Locally advanced or M1 with no evidence of disease after primary resection and metastasectomy completely resected <1 year from nephrectomy Intermediate-high risk	Adjuvant	Pembrolizumab	496	NR	0.68; 0.53–0.87; *p* = 0.001)	NR	0.54; 0.30-0.96			Any Grade: 96.3% G3–5: 32.4%
				Placebo	498	NR		NR				Any Grade: 91.1% G3–5: 17.7%

ORR = overall response rate; CR = complete response; PR = partial response; PFS = progression free survival; HR = hazard ratio; AE = adverse events.

#### 2.1.2. Investigational Approaches

*Perioperative ICI*

Several large phase 3 clinical trials are being performed to evaluate perioperative ICI in patients with intermediate or high-risk localized RCC. In the phase 3 KEYNOTE-564 trial, adjuvant pembrolizumab versus placebo demonstrated significant improvement with a hazard ratio for disease recurrence or death of 0.68, making it the first trial to show benefit for adjuvant immunotherapy in localized RCC [8]. Results from other studies are being awaited, including the phase 3 PROSPER Trial, in which nivolumab is given as neoadjuvant treatment followed by nine adjuvant doses after nephrectomy [57]. The phase 3 CheckMate 914 trial is evaluating adjuvant nivolumab and nivolumab plus ipilimumab versus placebo [58]. The IMmotion010 trial is evaluating adjuvant atezolizumab in a similar setting (NCT03024996) [59,60].

*Alternative Therapy Schedules*

An ongoing phase 2 trial is evaluating intermittent nivolumab dosing in which patients who achieve >10% reduction in tumor burden enter a treatment-free observation period and are re-imaged every 3 months. In this study, out of five patients who met criteria for treatment-free observation, only one patient re-started therapy and the other four sustained responses for median of 34 weeks after therapy discontinuation [61].

*Triple Therapy*

Triple combination cabozantinib with nivolumab and ipilimumab in clear cell RCC demonstrated tolerability and encouraging outcomes and is being evaluated in a multi-center, randomized phase 3 clinical trial (NCT03937219) [62].

#### 2.1.3. Biomarkers for ICI Treatment in RCC

There are no widely accepted models predictive of response and clinical benefit to ICI treatment in patients with RCC; however, prospective and retrospective analyses have identified demographic, clinical, histologic and molecular biomarkers which associate with treatment outcomes. 

Assocation between PD-L1 expression and outcomes to ICI treatment have been mixed in RCC. In the CheckMate 025 trial, patients found to have PD-L1 expression >1% had worse survival outcomes than those with low PD-L1 expression, even when treated with single agent anti-PD-1. This was not observed in the CheckMate 214 trial of combination anti-CTLA-4/PD-1 [44]. Interestingly, increased tumor cell PD-L1 was associated with shorter survival in patients treated with sunitinib or pazopanib in the COMPARZ trial in metastatic clear cell RCC, suggesting increased PD-L1 on tumor cells may represent a more clinically aggressive disease [63].

In the IMmotion150 trial of atezolizumab with or without bevacizumab versus sunitinib in metastatic RCC, gene expression profiling of pre-treatment tumor samples was found to associate with clinical outcomes within and across treatment arms [55]. Tumors with higher-than-median expression of a T cell effector gene signature (T_eff_ signature, T_eff_^High^) had improved ORR to atezolizumab plus bevacizumab treatment versus T_eff_^Low^. Similar benefit was observed in the JAVELIN Renal 101 trial, where higher-than-median Immuno Score resulted in more favorable outcomes in patients treated with avelumab plus axitinib [6,64]. T_eff_ and Immuno score did not meaningfully impact outcomes in VEGF TKI monotherapy arms. 

A myeloid-related inflammation signature was also profiled in both studies. Tumors with concurrent myeloid^High^ and T_eff_^High^ were less likely to benefit from atezolizumab monotherapy. Interestingly, in the IMmotion150 trial, benefit in patients with myeloid^High^ T_eff_^High^ tumors was preserved with combination atezolizumab plus bevacizumab, suggesting the addition of bevacizumab may overcome myeloid inflammation-associated resistance in these tumors. In the JAVELIN Renal 101 trial, patients with myeloid^High^ T_eff_^High^ tumors had worse outcomes in both avelumab plus axitinib and sunitinib treated arms.

VEGF-inducible angiogenesis-associated gene signature (Angio signature) associated with benefit to VEGF-TKI monotherapy. Patients with angio^High^ tumors experienced greater benefit from VEGF-TKI monotherapy while patients with Angio^Low^ had better outcomes when treated with ICI plus VEGF TKI [55,64,65]. This suggests utility of Angio score as a way to select patients for VEGF TKI monotherapy.

As mentioned above, IMDC favorable-risk score associated with inferior outcomes to nivolumab plus ipilimumab treatment [7]. In the IMmotion150 trial, benefit to atezolizumab plus bevacizumab was seen across IMDC risk groups [55]. An elevated BMI associated with improved outcomes to ICI treatment in RCC in two independent retrospective studies [20,21]. Association between neutrophil-to-lymphocyte ratio and outcomes has been mixed and larger prospective cohorts are needed to confirm its predictive role [20,66]. Lastly, retrospective analysis of multiple studies has found loss-of-function truncating *PBRM1* mutations may confer increased responsiveness to ICI [67,68,69]. However, recent evaluation in first-line setting did not corroborate these findings [55]. 

### 2.2. Bladder Cancer (Urothelial Carcinoma)

Bladder cancer, or urothelial carcinoma (UC), is the sixth most common cancer diagnosis among men and women in the United States. In 2020, an estimated 84,000 new cases of bladder cancer were diagnosed, with 75% of cases affecting men [70]. ICI has been approved for treatment of bladder cancer including both non-muscle-invasive and locally advanced or metastatic disease. Results from pivotal clinical trials are summarized in Table 3.

#### 2.2.1. ICI Indications

##### Non-Muscle-Invasive Bladder Cancer

Up to 40% of patients with high-risk non-muscle-invasive bladder cancer (NMIBC) will recur after Bacillus Calmette–Guerin (BCG) intravesicular immunotherapy for which radical cystectomy is indicated [71]. Many patients with urothelial cancer are elderly or of compromised performance status and cannot tolerate radical cystectomy, for which contemporary 90-day mortality estimates have been reported to be >10% [72]. As such, there is a need for treatment options for patients who cannot tolerate or wish not to undergo surgery. Pembrolizumab was recently evaluated in KEYNOTE-057, a multi-center, single-arm phase 2 clinical trial in patients with high-risk, BCG-unresponsive NMIBC who were ineligible for or elected not to undergo cystectomy. Pembrolizumab treatment resulted in a 41% complete response rate at 3 months assessed by cystoscopy, urine cytology and CT urography [73]. Encouragingly, 46% of patients who achieved response maintained a complete response at 12 months. This trial resulted in FDA approval in this setting.

##### Locally Advanced or Metastatic Bladder Cancer

*First Line*

In patients with metastatic bladder cancer, platinum-based chemotherapy (PBC) with cisplatin is preferred first-line standard of care with carboplatin as an alternative. ICI for first-line treatment in metastatic bladder cancer was first evaluated in cisplatin-ineligible patients. Results from the single-arm KEYNOTE-052 and IMvigor210 clinical trials of pembrolizumab and atezolizumab monotherapy in this setting resulted in accelerated approval by the FDA in 2017 [74,75]. Subsequent confirmatory phase 3 randomized clinical trials of these agents in the KEYNOTE-361 and IMvigor130, respectively, revealed first-line pembrolizumab and atezolizumab monotherapy did not improve outcomes versus standard of care PBC. This resulted in a label restriction of anti-PD-1 monotherapy for use only in cisplatin-ineligible patients whose tumors express PD-L1 (combined positive score ≥ 10), or in patients who are not eligible for any PBC (cisplatin or carboplatin) regardless of PD-L1 status [76,77]. Of note, in KEYNOTE-361, patients treated with pembrolizumab, responses were more durable compared to chemotherapy-containing arms. 

In addition to evaluating ICI monotherapy versus PBC, the KEYNOTE-361 and IMvigor130 clinical trials evaluated first-line ICI in combination with PBC, analogous to ICI-chemotherapy combinations used for treatment of non-small cell lung cancer. The KEYNOTE-361 trial found no improvement in outcomes with the addition of pembrolizumab to PBC versus PBC alone [76]. In the IMvigor130 trial, addition of atezolizumab to PBC prolonged PFS; however, improvement in OS did not reach statistical significance at interim analysis [77,78,79]. Combination treatment led to a near doubling of complete responses versus chemotherapy alone. In addition, data suggest cisplatin-treated patients derived greater OS benefit from addition of atezolizumab than carboplatin-treated patients; further follow-up is ongoing [80].

The DANUBE trial, a randomized phase 3 trial combining anti-PD-1 durvalumab and anti-CTLA-4 tremelimumab, did not result in survival benefit versus PBC [81].

*Second Line*

Multiple ICI, including pembrolizumab, atezolizumab, nivolumab, durvalumab and avelumab, received accelerated approval for treatment of patients who progressed on first-line platinum-based chemotherapy (PBC) or within 12 months of neoadjuvant or adjuvant PBC [82,83,84,85]. The confirmatory phase 3 KEYNOTE-045 clinical trial of pembrolizumab versus paclitaxel, docetaxel or vinflunine treatment confirmed improved objective response rate and overall survival [77,86]. However, the confirmatory phase 3 IMvigor211 clinical trial of atezolizumab monotherapy in relapsed bladder cancer failed to meet its primary endpoint of improving OS versus investigator’s choice of chemotherapy, and the company has since voluntarily withdrawn its indication for the refractory setting [87,88]. The indication for durvalumab has also been voluntarily withdrawn [89].

*Maintenance Therapy*

Results of randomized clinical trials of ICI confirmed a role for first-PBC in patients with metastatic bladder cancer. Use of ICI avelumab immediately after PBC induction (as maintenance therapy), rather than at progression, was evaluated in the JAVELIN Bladder 100 clinical trial in patients whom either achieved response or presented stable disease to first-line PBC. In this trial, avelumab maintenance was found to provide improved PFS and OS versus best supportive care (BSC) [9]. Importantly, the OS benefit for avelumab maintenance occurred after greater than 40% of the control group crossed over and received ICI after progression, revealing a benefit of starting ICI immediately after first-line chemotherapy rather than at disease progression.

**Table 3:** ICI clinical trials in urothelial carcinoma.

ICI Agent	Trial (Phase)	Setting	Line	Arms	N	ORR, CR, PR	Median PFS (months)	PFS HR (95% CI)	Overall Survival	OS HR (95% CI)	OS in PD-L1 Positive	AE
Atezolizumab	IMvigor130 (3) NCT02807636 [16,17]	Locally advanced or mUC * ECOG PS </= 2 Cisplatin-eligible or ineligible	1	A: Atezolizumab + carboplatin or cisplatin + gemcitabine	451	47%, 13%, 35%	8.2 (6.5–8.3)		16.0 (13.9–18.9)			% of patients AEs leading to treatment discontinuation 34%
				B: Atezolizumab monotherapy (Arm added after protocol amendment)	362	235, 6%, 17%			15.7 (13.1, 17.8)	HR B vs. C: 1.02 (0.83–1.24)	In pts with IC2/3 **: NE (Not evaluable)	6%
				C: Placebo + carboplatin or cisplatin + gemcitabine	400	44%, 7%, 37%	6.3	HR A vs. C: 0.82 (0.70–0.96) *p* = 0.007	13.4 (12.0, 15.2)	HR A vs. C: 0.83 (0.69–1.00) *p* = 0.027	In pts with IC2/3 **: 17.8 HR B vs. C: 0.68, (95% CI 0.43–1.08)	34%
Atezolizumab	IMvigor210 (2) [75]	Cisplatin-ineligible	1	Atezolizumab	123	23%, 9%	2.7 (2.1–4.2)		15.9 (10.4-NE)		OS by IC (PD-L1 status on immune cells): IC2/3: 12.3 (6.0-NE) IC0/1: 19.1 (9.8-NE)	G3–4: 16%
Pembrolizumab	Keynote-361 (3) NCT02853305 [76]	First-Line Locally advanced or mUC * ECOG PS </= 2 GFR > 30mL/min Randomized 1:1:1 >6 mo since MIBC ttr	1	A: Plat/gem + pembrolizumabMaintenance: Pembro	351	54.7%, 15.1%, 39.6%	8.3		17 (14.5–19.5)			Any G3–5: 87.4%
				B: Plat/gem Maintenance: none	352	44.9%, 12.2%, 32.7%	7.1	HR A vs. B: 0.78 (0.65–0.93) *p* = 0.0033	14.3 mo (12.3–16.7)	HR A vs. B: 0.86 (0.72–1.02) *p* = 0.0407	In pts with PD-L1 CPS >10 HR of A vs. B: 0.90 (95% CI 0.69–1.18)	Any G3–5: 81.9%.1%
				C: Pembrolizumab	307	30.3%, 11.1%, 19.2%	3.9 Due to statistical design, OS of B vs. C was not tested		15.6 (12.1–17.9)			Any G3–5: 62.9%
Pembrolizumab	Keynote-052 (2) NCT02335424 [74]	Cisplatin-ineligible	1	Pembrolizumab	370	24%, 5%, 19%					Response rate in PD-L1-expression of 10%: 42% (28–48%)	G3–4: 10%
Durvalumab	DANUBE (3) NCT02516241	Locally advanced or mUC * ECOG PS </= 2 Randomized 1:1:1	1	A: Plat/Gem	344	49%, 6%, 43%	6.7		12.1 (10.9–14.0)		OS in PD-L1 >25%, either tumor and immune cells 12.1 (10.4–15.0)	Grade 3–4 AE: 60%
Tremelimumab				B: Durvalumab + Tremelimumab	342	36%, 8%, 28%	3.7		15.1 (13.1–18.0)	HR A vs. B: 0.89 (0.71–1.11) *p* = 0.3039		Grade 3–4 AE: 27%
				C: Durvalumab	346	26%, 8%, 18%	2.3				14.4 (10.4–17.3) HR A vs. C: 0.85 (0.72–1.02) *p* = 0.0751	Grade 3–4 AE: 14%
Durvalumab	½ NCT01693562 [90]	Locally advanced or mUC	2	Durvalumab	191	17.8%, 3.6%, 14.2%	1.5 (1.4–1.9)				18.2 (81.-NR)	Grade 3–4 AE: 2%
Avelumab	Javelin Bladder 100 NCT02603432 [9]	mUC	1 Maintenance	A: Avelumab maintenance(treatment after platinum-based induction)	350				21.4		NE (20.3-NR)	All: 98% Grade 3–4: 47.4%
				B: Best supportive care	350				14.3	HR 0.69 (0.56–0.86) *p* = 0.001	17.1 (13.5–23.7) HR: 0.56 (0.4–0.79) *p* < 0.001	All: 77.7% Grade 3–4: 25.2%
ICI Agent	Trial (Phase)	Setting	Arms		N	Response Rate	DFS	DFS HR (95% CI)	In PD-L1 (+) Populations			AES
Pembrolizumab	PURE-01 (2) NCT02736266 [91]	Localized, muscle-invasive, M0 UC	Neoadjuvant: 3 courses preceding radical cystectomy	Pembrolizumab	27	pT0 Rate:42% (28.2–56.8%) pT < 2 = 54% (39.3–68.2%)			pT0 in PD-L1 CPS ≥ 10%: 54.3% pT0 in CPS < 10%: 13.3%			G3-4: 6%
Atezolizumab	ABACUS (2) NCT03800134 [92]	Localized, muscle-invasive, M0 UC	Neoadjuvant: 2 cycles prior to radical cystectomy	Atezolizumab	88	pT0 Rate: 31% (21–41%)			pT0 Rate in tumors with PD-L1 >5% of immune cells: 37% (21–55%)			G3-4: 12%
Atezolizumab	IMvigor010 (3) NCT02450331 [93]	Localized, muscle-invasive, M0 UC	Adjuvant: enrolled within 14 wees post-surgery, treated for up to 1 year	Atezolizumab	406		19.4 months (95% CI 15.9–24.8)	0.89 [95% CI 0.74–1.08]; *p* = 0.24				Serious AE: 31%
				Observation	403		16.6 months (11.2–24.8)					18%
Nivolumab	CheckMate-274 (3) NCT02632409 [10]	Localized, muscle-invasive, M0 UC	Adjuvant: after radical surgery +/− neoadjuvant cisplatin	A: Nivolumab	353		20.8 (16.5–27.6)		Median DFS in PD = L1 >/= 1% NR (21.2–NE)	63 (17.9)		>/=G3 17.9%
				B: Placebo	356		10.8 (8.3–13.9)	0.70 (0.57–0.86), *p* = 0.0008	8.4 (5.6–21.2) HR: 0.55 (0.39–0.77); *p* = 0.0005	25 (7.2)		>/=G3 7.2%

* mUC = metastatic urothelial carcinoma. ** PD-L1 staining of tumor-infiltrating immune cells (IC); IC2/3 indicates >5% PD-L1 expression.

#### 2.2.2. Investigational Approaches

*Enfortumab Vedotin Plus Pembrolizumab*

The antibody-drug conjugate enfortumab vedotin is being evaluated in combination with pembrolizumab as first-line therapy in locally advanced and metastatic bladder cancer. Initial results of the EV-103 phase 1b clinical trial revealed an encouraging response rate of 73% and reassuring safety profile [94]. Durability was demonstrated with a median duration of response of 25.6 months and approximately 53% of patients achieving responses lasting at least 24 months [95,96].

*Perioperative* *ICI*

There are multiple ongoing and completed clinical trials of ICI treatment in the perioperative setting in localized bladder cancer [97]. In the phase 3 placebo-controlled CheckMate 274 study, nivolumab given after radical cystectomy with or without neoadjuvant platinum based chemotherapy in patients with high-risk of recurrence demonstrated improved DFS in all patients independent of tumor PD-L1 status, although greater benefit was seen in patients with PD-L1 expression >1% [10]. This study resulted in FDA approval for nivolumab for adjuvant treatment of urothelial carcinoma independent of prior neoadjuvant chemotherapy, nodal involvement or PD-L1 status [98]. The PURE-01 and ABACUS clinical trials evaluated neoadjuvant single-agent pembrolizumab and atezolizumab, respectively, and demonstrated feasibility and tolerability with minimal delays in surgery and an encouraging rate of pathologic complete responses [91,92]. In the phase 3 IMvigor010 trial, adjuvant atezolizumab failed to meet its primary endpoint of improving OS compared with observation [93]. Combination ICI or single agent ICI plus chemotherapy are being evaluated in phase 3 trials [99,100]. Preliminary activity for neoadjuvant ICI in bladder cancer with variant histology has also been reported [101]. Long-term survival benefit and cost effectiveness analysis remain to be reported. 

*IO and Bladder Preservation Approaches*

In patients with locally advanced muscle-invasive bladder cancer, multi-modal bladder preservation treatment strategies are being investigated as an alternative to cystectomy. As mentioned above, cystectomy carries high morbidity and mortality and there is a need for alternatives to surgery. A phase 2 study of anti-PD-1 durvalumab plus anti-CTLA-4 tremelimumab with concurrent radiation therapy demonstrated feasible and encouraging outcomes [102]. Pembrolizumab in combination with gemcitabine and concurrent radiation therapy also demonstrated good tolerability and encouraging efficacy [103]. Two ongoing phase 3 clinical trials, S1806 and KEYNOTE-992, are evaluating concurrent chemoradiotherapy with or without atezolizumab and pembrolizumab in MIBC, respectively (NCT03775265, NCT04241185).

#### 2.2.3. Biomarkers for ICI Treatment in Bladder Cancer

*PD-1/PD-L1 Status*

No consensus has been reached on the prognostic value of PD-L1 expression in bladder cancer as its association with clinical benefit has been inconclusive [104,105]. A positive correlation between PD-L1 expression on immune cells (IC), but not tumor cells (TC), and response to anti-PD-1/L1 ICI has been observed in the second line setting [83,106]. A combined IC/TC score, the CPS score, defined as the percentage of tumor cells and infiltrating immune cells with positive PD-L1 expression of the total number of tumor cells, provides greater positive and negative predictive value than TC or IC evaluation alone. However, the CPS score was not associated with improved OS to ICI monotherapy versus chemotherapy in the KEYNOTE-361 trial [90,107].

In the phase 3 IMvigor130 trial, improved interim OS was seen in patients with PD-L1 IC2/3 (>/=5% PD-L1 expression on immune cells via Ventana SP142 IHC) treated with atezolizumab monotherapy versus PBC [84]. In addition, patients with concomitant TMB^High^ and PD-L1 IC2/3 had more profound benefit to atezolizumab monotherapy.

Currently, a positive PD-1/L1 status is an indication to use anti-PD1/PD-L1 monotherapy in patients who cannot receive cisplatin [79]. If the patient is ineligible for any platinum-containing chemotherapy, atezolizumab or pembrolizumab are indicated in the first line setting regardless of PD-L1 status.

*Other Biomarkers*

Higher than median expression of a T cell-effector gene expression signature has not consistently associated with benefit to ICI in large clinical trials in metastatic bladder cancer [70,108]. Notably, in the phase 3 IMvigor130 trial, a positive T cell-effector gene expression signature did not correlate with improved OS in atezolizumab containing arms versus PBC alone.

Gene expression signatures that profile TGF-β signaling, such as the fibroblast TGF-β-response signature (F-TBRS) or the stromal/EMT/ TGF-β score, have been linked to extracellular matrix (ECM) dysregulation and T cell exclusion. These genes expression signatures have associated with worse response to ICI in metastatic bladder cancer, including the IMvigor130 trial and KEYNOTE-052, where patients with higher median F-TBRS gene expression and high stromal/EMT/ TGF-β score had inferior OS to treatment with atezolizumab and pembrolizumab monotherapy, respectively [108,109,110,111,112]. Associations between inferior clinical outcomes and expression of TGF-β-induced genes were also observed in the ABACUS trial of neoadjuvant atezolizumab [92]. 

APOBEC mutagenesis gene expression signature was found to correlate with improved overall survival in atezolizumab containing arms in the IMvigor130 study [108].

Bladder cancer has one of the highest somatic mutations rates and TMB status correlates with response to ICI [106,113]. In addition, mutations in DNA damage response (DDR) genes have associated with clinical benefit in ICI in bladder cancer [114]. However, in the ABACUS trial of neoadjuvant atezolizumab, TMB or mutations in DDR genes did not associate with improved outcomes [92].

Analysis of samples from a phase 2 trial of atezolizumab revealed low expression of endoplasmic reticulum aminopeptidase 2 (ERAP2) associated with improved survival to ICI [106,115]. 

### 2.3. Prostate Cancer

Over the past decade, both anti-CTLA4 and anti-PD1/PD-L1 agents have been studied extensively in metastatic castrate-resistant prostate cancer (mCRPC); however, no ICI or ICI-based combination evaluated to date has received FDA approval (Table 4). Examination of prostate tissues acquired from cystoprostatectomy in advanced bladder cancer patients treated with neoadjuvant ipilimumab revealed favorable immunologic changes in malignant and non-malignant prostate tissues [116]. Ipilimumab in combination with radiotherapy was initially evaluated in the CA184-043 clinical trial, a multi-center, randomized phase 3 trial in patients with mCRPC who progressed after docetaxel chemotherapy. Ipilimumab plus radiotherapy failed to meet its primary endpoint of improving OS; however, recent pre-planned long-term analysis signaled OS benefit for ipilimumab-treated patients [117,118]. The phase 3 CA184-095 clinical trial evaluated ipilimumab in chemotherapy-naïve mCRPC patients and excluded patients with visceral metastases; however, ipilimumab failed to improve OS versus placebo [119]. Failure to reach primary endpoint in this study was impacted by improved survival in the control arm versus historical controls, suggesting improvement in standard of care for the treatment of mCRPC patients. Despite negative results, subsequent studies have observed clinical benefit in a subset of patients treated with ipilimumab. A non-randomized phase 2 study of ipilimumab in mCRPC demonstrated that patients with evidence of pre-existing immunity on pre-treatment samples, such as a high intratumoral CD8 T cell density or IFN-γ response gene signature, had favorable outcomes to treatment [120,121]. 

Ipilimumab in combination with nivolumab was evaluated in the phase 2 CheckMate 650 trial and demonstrated encouraging response rates in mCRPC patients both pre- and post-chemotherapy including complete responses [121]. This trial has recently begun to accrue additional patients.

Anti-PD-1/PD-L1 agents as monotherapy have been studied in mCRPC but have provided minimal benefit. Pembrolizumab, evaluated in a multi-cohort, non-randomized study in patients with mCRPC resulted in minimal response [122]. The KEYNOTE-199 clinical trial, a multi-cohort phase 2 clinical trial of pembrolizumab monotherapy in previously treated patients with PD-L1-positive, PD-L1-negative and bone-predominant disease showed only modest anti-tumor response in all cohorts [123]. 

Anti-PD-1/PD-L1 agents have been evaluated in combination with chemotherapy, novel anti-androgens and other agents. Atezolizumab in combination with novel anti-androgen enzalutamide did not improve OS compared with enzalutamide alone in a large phase 3 clinical trial [124]. Atezolizumab in combination with cabozantinib resulted in encouraging ORR in pre-treated population in the Phase Ib COSMIC-021 study [125]. A phase 3 study of pembrolizumab plus docetaxel plus prednisone has recently been initiated [126]. There is an ongoing phase 3 clinical trial evaluating combination pembrolizumab plus olaparib versus novel anti-androgen monotherapy in mCRPC [127].

There are no FDA approved indications for ICI for treatment of castrate-sensitive prostate cancer (mCSPC); however, their use is being evaluated in clinical trials. A phase 3 trial is underway to evaluate pembrolizumab plus enzalutamide plus androgen deprivation therapy (ADT) versus enzalutamide and ADT alone [NCT04191096]. Multiple phase 1 and phase 2 trials are evaluating ICI in combination with treatments such as abiraterone and cabozantinib [NCT04477512], radiation therapy [NCT04262154, NCT03795207] and an experimental IL-8 directed monoclonal antibody [NCT03689699]. In addition, perioperative ipilimumab in combination with castration prior to radical prostatectomy has demonstrated feasibility with longer follow-up ongoing [128].

#### Biomarkers for ICI Treatment in Prostate Cancer

Biomarker-directed treatment selection has become standard of care in prostate cancer. Two to five percent of prostate cancers have been found to have microsatellite instability-high status for which pembrolizumab has recently been approved in the refractory setting [16,129,130]. In mCRPC with mutations in BRCA or other homologous recombination DNA-damage repair genes, PARP inhibitors, such as rucaparib, have been recently approved [131,132]. Pre-clinical work has suggested PARP inhibitor treatment may activate the cGAS-STING pathway through accumulation of cytosolic double-strand DNA breaks and prime anti-tumor immunity and increase therapeutic efficacy of ICI independent of BRCA status [133]. This observation led to clinical trials evaluating anti-PD-1/PD-L1 treatment in combination with PARP inhibitors in unselected population of mCRPC patients; however, these trials have demonstrated inconsistent benefit [134,135].

A single arm phase 2 study of ipilimumab in mCRPC found the presence of tumor-infiltrating lymphocytes by IHC and IFN-γ gene signatures associated with favorable outcomes [120]. 

Loss-of-function alterations of tumor suppressor protein CDK12, found in approximately 5–7% of prostate cancers, results in genomic instability, increased neoantigen burden and T cell infiltration [136,137]. Retrospective analyses of heavily pre-treated patients with CDK12 mutated prostate cancer observed clinical activity of anti-PD-1 ICI suggesting CDK12 may serve as a biomarker for ICI response [138]. Recent correlate analysis of mCRPC biopsies revealed CDK12-mutated mCRPCs were enriched in immunosuppressive CD4 + FOXP3- cells [139].

**Table 4:** ICI clinical trials in prostate adenocarcinoma.

ICI Agent	Trial (Phase)	Setting	Arms	N	ORR	Median PFS	PFS HR (95% CI)	Overall Survival	OS HR (95% CI)	OS in PD-L1 Positive	AE
Ipilimumab	CheckMate650 (2) NCT02985957 [121]	mCRPC Cohort 1 (*n* = 45): progressed after >1 2nd gen hormone therapy, chemotherapy naïve	Ipilimumab (3mg/kg) + Nivolumab for up to four doses then nivolumab monotherapy	45	25%	5.5 mo (3.5–7.1)		19.0 mo (11.5-NE)			G3–4: 42.2%
Nivolumab		Cohort 2 (*n* = 45): progressed after chemotherapy		45	10%	3.8 mo (2.1–5.1)		15.2 mo (6.4-NE)			G3–4: 53.3%
Ipilimumab	CA184-095 (3) NCT01057810 [119]	mCRPC Chemotherapy naïve Asymptomatic or minimally symptomatic Without visceral metastases	Ipilimumab (10 mg/kg)	400	PSA Response * 23% (19–27%)	5.6 mo		28.7 mo (24.5–32.5)			G3–4: 15%
			Placebo	202	8% (5–13%)	3.8 mo	HR: 0.67 (0.55–0.81)	29.7 mo (26.1–34.2)	HR: 1.11; (0.88 to 1.39) *p* = 0.3667		G3–4: 1%
Ipilimumab	CA184-043 (3) NCT01057810 [117,118]	mCRPC Post-docetaxel	Ipilimumab (10 mg/kg) + Radiation Therapy	399				11.2 mo (9.6–12.6)			G3–4: 58.7%
			Placebo + Radiation Therapy	400				10.0 mo (8.4–11.2)	HR: 0.84 (0.72–0.98), *p* = 0.03 * at 2-year minimum follow up		G3–4: 41%
Pembrolizumab	Keynote-199 (2) NCT02787005 [123]	mCRPC Cohort 1: PD-L1 positive	Pembrolizumab	133	5% (2–11%)			9.5 mo			G3–4: 15%
		Cohort 2: PD-L1 negative		66	3% (<1–11%)			7.9 mo			
		Cohort 3: Bone-predominant		59	NE			14.1 mo			
Atezolizumab	IMbassador250 (3) NCT03016312 [124]	mCRPC Progressed on abiraterone and docetaxel	Atezolizumab + enzalutamide	379				15.2 mo (14.0–17.0)			G3–4: 28.3%
			Enzalutamide	380				16.6 mo (14/7–18.4)	HR, 1.12 (0.91, 1.37), *p* = 0.28		G3–4: 9.6%

* PSA response = 50% decrease from baseline confirmed by a second PSA value >/= 6 weeks later.

### 2.4. Testicular Cancer

Testicular germ cell tumors (TGCTs) are highly sensitive to cisplatin-based chemotherapy and many achieve cure with either upfront or salvage chemotherapy upon relapse. In chemotherapy-refractory cases, ICI has been studied in multiple case series as well as early phase clinical trials [140,141,142]. Single-arm phase 2 clinical trials of anti-PD-1/PD-L1 inhibitors pembrolizumab, durvalumab and avelumab did not demonstrate meaningful responses in refractory TCGTs [138,143,144]. Low response rates have been attributed to the immunosuppressive microenvironment of testicular tissue. TGCTs have been observed to have a low tumor mutational burden and have varying expression of PD-1/PD-L1 [145,146].

## 3. Biomarkers for ICI Treatment

While durable responses to ICI treatment have been observed in multiple cancer types, only approximately 15–30% patients achieve response in aggregate [147,148]. Correlative analysis of clinical trial samples has identified features of the tumor cells, referred to as tumor-intrinsic features, and of the TME, or tumor-extrinsic features, which reflect prognosis or predict response to ICI. These biomarkers include histopathologic and molecular features as well as demographic and clinical factors.

With rapidly evolving treatment options and clinical trial landscape within GU malignancies, development and incorporation of prognostic and predictive biomarkers is critical to personalize treatment selection and optimize clinical benefit for patients. Large clinical trials of ICI in GU malignancies and other solid tumors have prospectively evaluated the predictive and prognostic value of histopathologic and molecular assessment of individual tumor characteristics and the TME. Some of these factors have been incorporated into guideline-based approvals of ICI. Herein, we review established and investigational biomarkers being utilized in the current landscape of ICI with a focus on GU malignancies.

### 3.1. The Immune Contexture: Characteristics of the Tme

Tumor-promoting inflammation and avoidance of immune destruction are considered hallmarks of cancer [149]. Characteristics of a host’s immune response to tumor development, referred to as the immune contexture, have an increasingly recognized impact on clinical outcomes. For example, type, location, and density of immune cells within the TME of colorectal tumors were found to correlate strongly with survival [150,151]. 

The presence of infiltrating T cells and supporting pro-inflammatory milieu, defined as the T cell-inflamed tumor microenvironment (TME), has associated with improved clinical outcomes in patients treated with ICI in a variety of solid tumors [2,13]. In contrast to T cell-inflamed TME, tumors may lack infiltrate, referred to as immune deserts, or be characterized by immune exclusion, where T cells are found on the tumor edge [152]. In GU malignancies, a high proportion of RCC are found to be T cell inflamed at diagnosis while bladder cancer often has an immune-excluded phenotype. Prostate cancer often lacks an immune infiltrate altogether [153,154,155].

Factors associated with the development of T cell-inflamed TME remain areas of active research. To date, the field has recognized the importance of the presence of tumor-associated neoantigens sufficient to stimulate tumor-reactive T cells, the presence of antigen-presenting dendritic cells and a TME hospitable for accumulation of anti-tumor immune cells [153,156,157]. 

#### 3.1.1. Immunofluorescence/Immunohistochemical Analysis

Advances in multiplex immunofluorescence (IF) and immunohistochemical (IHC) techniques have allowed for spatial interrogation of a broad scope of immune cell populations within the TME of solid tumor malignancies. These techniques represent important modalities to appropriately characterize complex immune TME and their relation to prognosis and treatment outcomes. During initial evaluation of anti-PD-1 agents in melanoma, IHC characterization of CD8 positivity and PD-L1 expression were significantly associated with response to ICI [2]. CD8+ T cell density within tumor infiltrate has negatively associated with prognosis in prostate cancer and RCC, and positively associated in bladder cancer [158]. In one study in RCC, CD8+ T cell quantification on anti-PD-1 treated RCC samples found a majority of samples (73%) were infiltrated; however, infiltration did not correlate with PFS [69]. In another study, an 11-marker multiplex IF panel-based prognostic score associated with survival of RCC patients, particularly the density of T cell marker CD3-positive cell populations [159]. Interestingly, spatial immune profiling of nephrectomy samples revealed patients with excluded TME had reduced burden of metastatic disease and improved overall survival versus T cell-inflamed TME [160]. 

#### 3.1.2. Gene Expression Signatures

Quantification of RNA expression of gene subsets in tumor samples can characterize aspects of underlying tumor and TME immune biology and serve as predictive biomarkers for ICI treatment. Gene expression signature analysis of IFN-γ associated genes, cytotoxic T cell effector genes, dendritic cell and antigen presentation associated genes and T cell chemo-attractants have been shown to correlate with T cell infiltration and clinical benefit to ICI and cancer vaccines [2,14,161,162,163]. Similar T-cell inflamed gene expression signatures correlated with response to ICI in large-scale trials in RCC and bladder cancer [55,70]. However, many patients with higher-than-median expression of T cell-inflamed gene expression signature do not respond to ICI.

Certain gene expression signatures designed to assess activity of a specific biologic or signaling pathway, such as the angiogenesis signature or TGF-β signature have also been evaluated in pre-treatment tumor samples in large clinical trials of ICI in RCC and mUC, respectively [55,164]. These gene expression signatures may assist in identifying potential mechanisms of resistance to ICI and patients who may not respond to upfront ICI. For example, higher-than-median expression of a myeloid inflammation gene signature comprising cytokines and chemokines associated with recruitment of myeloid derived suppressor associated with worse outcomes to ICI monotherapy in RCC despite presence of concomitant positive T cell-inflammation [55]. Of note, VEGF-R blockade in pre-clinical models has been shown to mitigate these signaling pathways, suggesting a potential biomarker-directed indication for addition of VEGF-R blockade to ICI [165,166]. As discussed above, addition of bevacizumab to atezolizumab preserved benefit in patients with myeloid^High^ tumors. Alternatively, gene expression signatures which detect the presence of alternative immune checkpoints, such as LAG3 or TIM3, may inform selection of combination treatments.

Non-T cell-inflamed tumors identified by gene expression analysis may be tumors for which innate immune-stimulating agents, such as STING and TLR agonists, could stimulate T cell-inflammation and induce response to ICI [167]. Serial assessment of T cell-inflammation gene expression signatures, while on-treatment, may help assess efficacy of novel treatment strategies.

Use of gene expression signatures as predictive biomarkers has thus far been limited to prospective correlative analysis. As gene expression signatures continue to mature, incorporation into both FDA-approved indications may assist in optimal personalized treatment choices. In addition, incorporation of gene expression signatures into clinical trial inclusion criteria will enable optimal identification of patients for appropriate clinical trials and accelerate the development of new, personalized therapies [168]. 

#### 3.1.3. PD-L1 Expression

No consensus has been reached on the predictive value of immunohistochemical assessment of PD-L1 expression and response to ICI in GU malignancies. In large trials in mUC, PD-L1 expression by immune cells, not tumor cells, correlated with response. In RCC, PD-L1 expression did not correlate with improved outcomes in ICI plus VEGF TKI combination treatment [7,64,77,169]. In prostate cancer, only a 2% increase in response rate to pembrolizumab was observed between PD-L1 high versus low patient cohorts [123]. As predictive biomarkers continue to mature, it is likely that PD-1/PD-L1 status will continue to be utilized but likely in combination with other biomarker assessments.

#### 3.1.4. Tertiary Lymphoid Structures

Tertiary lymphoid structures, organized lymphoid aggregates made up primarily of B cells, have been identified within tumors of a broad range of malignancies. TLS quantification by H&E and immunohistochemical detection of B cells (CD20), T cells (CD3), follicular dendritic cells (CD21) and high endothelial venules (MECA79) revealed increased TLS associated with response to ICI in melanoma and RCC [170,171]. In bladder cancer, transcriptomic assessment CXCL13, a chemokine critical for TLS formation, was found to be associated with improved survival in ICI-treated bladder cancer [172].

#### 3.1.5. MicroRNA Signatures

MicroRNA are small single-stranded non-coding RNA nucleotides which negatively regulate gene expression by post-transcriptional RNA silencing. Analysis of human cancers has identified miRNA signatures which associate with tumor behavior and clinical outcomes to treatment [173]. Expression of certain miRNAs has correlated with absence of benefit to ICI treatment in patients with melanoma [174]. In a subset of patients with RCC treated with nivolumab, miRNAs which target genes involved in PI3K-Akt and T cell receptor signaling were found to be elevated in peripheral lymphocytes in patients who had durable responses to nivolumab [175].

### 3.2. Tumor-Intrinsic Properties

Tumor-intrinsic factors such as high somatic tumor mutation burden (TMB), defective mismatch repair pathways (dMMR) and dysregulated oncogenic signaling pathways have been shown to impact response to ICI.

#### 3.2.1. Defective Mismatch Repair

Tumors with defective genes associated with mismatch repair mechanisms (dMMR), such as MSH2, MSH6 and MLH1, or other DNA proofreading mechanisms have been shown to be sensitive to ICI [16,176]. Mechanistic basis of this sensitivity to ICI is postulated to be due increased mutational load resulting in immune cell infiltration [177,178]. In 2017, pembrolizumab was approved for treatment of metastatic dMMR or MSI-high positive solid tumors which had progressed on prior therapy and did not have alternative treatment options based on a non-randomized single arm study of around 200 patients [179]. Prevalence of dMMR is highly variable across tumor types and is highest in Lynch syndrome-associated tumor types (endometrial, colon, gastric and rectal) [180]. MSI-H status in GU malignancies is roughly 3% of prostate cancer patients, 2% of mUC and 1% of RCC [129,181,182].

#### 3.2.2. Tumor Mutation Burden

Tumors with high somatic mutation burden (TMB) have been found to have improved clinical outcomes to ICI across cancer types in part due to the association of TMB with neoantigen load and immune cell infiltration [17]. Pembrolizumab monotherapy is FDA approved in patients with refractory solid tumors with TMB ≥ 10 mutations/Mb, based on results from the phase 2 KEYNOTE-158 clinical trial [179]. Of note, small numbers of GU malignancies were included in this study. Recent analysis found that only certain tumor types have correlation between TMB, neoantigen load and CD8+ T cell infiltration [183]. Improved clinical outcomes to ICI were observed only in the cancer types where there was correlation, which included mUC but not RCC and prostate cancer. This observation is supported by clinical experience in clinical trials in GU malignancies in which TMB status associated with clinical benefit in bladder cancer but not in RCC [55,64,70]. A small phase 2 study of ipilimumab in mCRPC also corroborated this finding; however, this same study noted that T cell infiltration was observed in patients with relatively low TMB, suggesting certain mutational profiles may generate antigen-specific T cell responses independent of TMB status [120].

#### 3.2.3. Tumor and Treatment Characteristics

Increased tumor burden has correlated with worse outcomes to ICI treatment in solid tumors [184]. In addition, presence of liver metastasis is associated with worse clinical outcomes in patients with GU malignancies treated with ICI [185].

The association between occurrence of immune-related adverse events (irAEs) and outcomes has been mixed. In patients with metastatic melanoma treated with ipilimumab, studies found conflicting associations between development and an irAE with duration of response [186,187]. In RCC, small retrospective analyses have found association between irAE and clinical benefit to ICI [20,188,189]. Given these conflicting results, studies have begun to evaluate the differential impact on response to ICI by the organ system affected by irAEs. One study demonstrated certain organ involvement, thyroid for example, associated with favorable outcomes versus others that did not show a clear trend or were associated with worse prognosis [190,191].

### 3.3. Demographic and Clinical Factors

Patient demographic and clinical factors such as germline polymorphisms, gender, BMI and circulating factors have been shown to associate with response to ICI. Mechanistic exploration of each of these factors remains an active area of investigation.

#### 3.3.1. Germline Polymorphisms

Gene expression analysis has demonstrated how genes related to immune response are under strong germline genetic control suggesting an individual’s anti-tumor immune response is, in part, determined by one’s genome [115]. In addition, germline variants have been found to have a strong influence on immune cell production and response to cytokines [192]. A particular germline polymorphism resulting in low level expression of *ERAP2* associated with enhanced immunogenicity and improved survival in ICI-treated patients with luminal subtype of bladder cancer. In RCC, although somatic *PRBM1* mutations correlated with T cell infiltration, the infiltrated tumors were enriched for deleterious *PRBM1* mutations as compared to non-infiltrated tumors, which negatively impacted survival [69].

#### 3.3.2. Circulating Factors

Peripheral blood analysis represents a less-invasive method of personalized prediction and prognostication. In RCC and bladder cancer, a higher neutrophil-to-lymphocyte ratio associates with worse outcomes to ICI treatment [191,192]. Circulating tumor DNA (ctDNA) can identify genomic features of tumors which may predict ICI responsiveness [193]. For example, prognostic value of ctDNA-based TMB assessment is being evaluated in ongoing clinical trials [NCT02542293]. In addition, ctDNA has demonstrated utility as a surrogate for disease burden to assist in treatment decisions and need for adjuvant therapy after surgical resection or induction chemotherapy [194,195]. 

T cell receptor repertoire has been found to predict response to ICI. In one study, increased peripheral T cell receptor diversity associated with improved outcomes to ICI treatment in melanoma [196]. In contrast, less diversity of TCR in tumor-infiltrating lymphocytes associated with response to ICI, demonstrating the importance of both clonal expansion and diversity of T cells in response to ICI [2].

#### 3.3.3. BMI

Patients with elevated BMI were found to have improved outcomes to ICI in retrospective analysis of large clinical trials in RCC and metastatic melanoma, but other studies have reported mixed results [20,21,22,197]. It is well established that obesity is associated with dysregulated inflammatory processes which contribute to both tumorigenesis and impaired immune responses; however, precise mechanisms of impact on ICI treatment remain an area of active investigation [198,199,200]. Mechanistic studies in a diet-induced obesity preclinical model revealed obese mice had heightened tumor progression and associated immune dysfunction but improved response to ICI, suggesting that obesity may mediate pathways of tumorigenesis and immune activation, which render a tumor particularly susceptible to ICI [201]. In human RCC samples, transcriptomic analysis found patients with obese BMI did not have a higher degree of intra-tumoral immune cell infiltration but rather higher degrees of inflammation in peritumoral adipose tissue that may act as an immune cell reservoir to augment response to ICI [202]. In contrast, another study demonstrated obese patients with RCC had worse clinical outcomes with ICI treatment and decreased frequency of tumor-infiltration PD-1 expressing T cells in pre-treatment biopsies, which was recapitulated in a murine model [197]. Continued research is warranted. BMI is also being recognized as an imperfect measure of body composition and application of radiographic techniques such as computerized tomography-based composition has begun to further define fat types and muscle densities and outcomes to ICI [203,204].

#### 3.3.4. Commensal Microbiota

The commensal microbiome, which includes the gut and urinary microbiome, is known to have significant influence on tumorigenesis and the immune system, and there is a growing body of evidence demonstrating the impact of commensal microbiome composition on response to ICI [19,205,206,207,208]. Preclinical studies demonstrated fecal transfer between responder to non-responder mice could increase responsiveness to ICI via enhanced dendritic cell-mediated T cell activation [19,209]. Recent prospective analysis of RCC patients treated with ICI revealed association between gut microbial diversity and benefit to treatment [210]. In non-muscle invasive bladder cancer, composition of the urinary microbiome was associated with response to BCG immunotherapy [211]. Manipulation of the microbiome through fecal microbiota transplant can influence efficacy of ICI and this is being evaluated in clinical trials [212,213]. Fecal microbiota transplantation has also been shown to effectively treat refractory ICI-associated colitis [214].

#### 3.3.5. Diet

Understanding the impact of diet and nutrient availability within the TME on immunometabolism and function of tumor and immune cells is also an area of active investigation. Studies of the ketogenic diet (KD), a high-fat, low carbohydrate diet has been shown to cause metabolic alterations in tumor cells and restrict tumor growth and sensitize tumors to chemotherapy and radiotherapy [215,216,217]. Recent research has shown KD may impact immune cell populations including an increase in favorable T cell populations in adipose tissue [218]. Intermittent KD diet in mice was shown to facilitate T cell-mediated tumor control by preventing upregulation of PD-L1 on myeloid cells while promoting anti-cancer T cells [219]. Interestingly, a murine colorectal cancer model revealed high-fat diet (HFD) with similarities to KD increases tumor growth and reduces intratumoral T cell infiltration and activation [220]. Single-cell RNA sequence analysis revealed HFD can mediate opposing metabolic changes in tumor and immune cells within the TME resulting in immune dysfunction, specifically tumor cell upregulation of fatty acid oxidation (FAO) and reduced proliferative capacity of T cells. Currently, it is difficult to draw conclusions on the net effect of diets on cancer, and well-designed clinical trials are needed to assess their impact. Many clinical trials are underway across multiple tumor types.

## 4. Conclusions

Immune checkpoint inhibitors have become standard of care for the treatment of GU malignancies in both localized and metastatic disease settings. In patients with metastatic disease, continued emphasis must be placed on the development of novel ICI and ICI-based combinatorial strategies to increase response rates and enhance depth of response. In localized disease, further clarification of the impact of adjuvant ICI on surgical outcomes or as an alternative to surgery is awaited. Lastly, continued validation of biomarker-based approaches will be critical to personalize treatment strategies and optimize treatment efficacy and clinical benefit for patients.

## Figures and Tables

**Table 1:** Immune checkpoint inhibitors in current clinical use.

Immune Checkpoint Inhibitor	Target
Atezolizumab (Tecentriq)	PD-L1
Avelumab (Bavencio)	PD-L1
Cemiplimab (Libtayo)	PD-1
Durvalumab (Imfinzi)	PD-L1
Ipilimumab (Yervoy)	CTLA-4
Nivolumab (Opdivo)	PD-1
Pembrolizumab (Keytruda)	PD-1
Tremelimumab	CTLA-4
